# Over-expression of oncigenic pesudogene DUXAP10 promotes cell proliferation and invasion by regulating LATS1 and β-catenin in gastric cancer

**DOI:** 10.1186/s13046-018-0684-8

**Published:** 2018-01-27

**Authors:** Yongcan Xu, Xiang Yu, Chenchen Wei, Fengqi Nie, Mingde Huang, Ming Sun

**Affiliations:** 1 Department of General Surgery, Huzhou Central Hospital, Huzhou, People’s Republic of China; 2 Department of General Surgery, The Affiliated Yantai Yuhuangding Hospital of Qingdao University, Yantai, People’s Republic of China; 3 Department of Oncology, Second Affiliated Hospital, Nanjing Medical University, Nanjing, People’s Republic of China; 4 Department of Oncology, First Affiliated Hospital, Nanjing Medical University, Nanjing, People’s Republic of China; 5 Department of Oncology, Huai’an First People’s Hospital, Nanjing Medical University, Huai’an, People’s Republic of China; 6 Department of Bioinformatics and computational biology, UT MD Anderson Cancer Center, 1400 Pressler Street, Unit 1410, Houston, TX 77030 USA

**Keywords:** Pesudogene, DUXAP10, Gastric cancer, Aggressive phenotype, Prognostic biomarker

## Abstract

**Background:**

Recently, the pesudogenes have emerged as critical regulators in human cancers tumorigenesis and progression, and been identified as a key revelation in post-genomic biology. However, the expression pattern, biological function and mechanisms responsible for these molecules in human gastric cancer (GC) are not fully understood.

**Methods:**

In this study, we globally assessed the transcriptomic differences of pesudogenes in gastric cancer using publicly available microarray data. DUXAP10 expression levels in GC tissues and cells was detected using quantitative real-time PCR (qPCR). DUXAP10 siRNAs and over-expression vector were transfected into GC cells to down-regulate or up-regulate DUXAP10 expression. Loss- and gain-of function assays were performed to investigate the role of DUXAP10 in GC cells cell proliferation, and invasion. RIP, RNA pulldown, and ChIP assays were used to determine the mechanism of DUXAP10’s regulation of underlying targets.

**Results:**

The pesudogene DUXAP10 is the only pseudogene that significantly over-expressed in all four GEO datasets, and frequently over-expressed in many other cancers including Liver Hepatocellular carcinoma, Bladder cancer, and Esophageal Cancer. High DUXAP10 expression is associated with GC patients poor prognosis, and knockdown of DUXAP10 significantly inhibits cells proliferation, migration and invasion in GC. Mechanistic investigation shows that DUXAP10 can interact with PRC2 and LSD1 to repress LATS1 expression at transcriptional level, and bind with HuR to maintain the stability of β-catenin mRNA and increase its protein levels at post-transcriptional level.

**Conclusions:**

Overall, our findings illuminate how increased DUXAP10 confers an oncogenic function in GC development and progression that may serve as a candidate prognostic biomarker and target for clinical management of GC.

**Electronic supplementary material:**

The online version of this article (10.1186/s13046-018-0684-8) contains supplementary material, which is available to authorized users.

## Background

Gastric cancer is one of the leading causes of cancer related death worldwide, and is the most common gastrointestinal malignancy in East Asia [1, 2]. In spite of the improvement in surgical techniques and targeted drug chemotherapy, the five-years overall survival rate remains unsatisfactory due to lots of patients were diagnosed at an advanced stage accompanied by lymphatic metastasis that limit the successful therapeutic strategies [3]. Although there are a great advancement on the gastric carcinogenesis, the molecular mechanisms underlying gastric cancer progression and tumor metastasis are still poorly understood [4]. Therefore, better understanding of the tumorgenesis and cancer cells metastasis is essential for the development of diagnostic markers and novel effective therapies for gastric cancer patients.

Benefiting from the advancement on bioinformatics and next generation sequencing technique, the completion of the ENCODE (Encyclopedia of DNA Elements) project and FANTOM (Functional Annotation of Mammals) consortia has highlighted the prevalence of non-protein coding functional elements in human genome [5, 6]. Recently, GENCOD release data showes that there are 60,498 genes (Version 23, March 2015 freeze, GRCh38), but only 19,797 of them are defined as protein-coding genes while the other genes are classified as small non-coding RNA, long non-coding RNA genes and pseudogenes. To date, numerous lines of evidence has demonstrated that microRNAs and lncRNAs play critical roles in the process of carcinogenesis and tumor progression [7–10]. However, pseudogenes have been regarded as ‘junk genes’ or biologically inconsequential due to the harbour mutations that abrogate their transcription or translation [11]. Interestingly, increasing evidence reveales the multilayered biological function of pseudogenes in diverse cellular processes, especially their contribution to human cancers by acting as endogenous competitors for miRNA, generating endogenous small-interference RNA (endo-siRNA or esiRNA) or through competing for RNA-binding protein (RBP) or translational machinery [12, 13].

Previously studies have demonstrated that many pseudogenes contribute to tumorigenesis through functioning as ceRNA. For example, the pseudogene PTENP1 possess a regulatory function of PTEN through acting as a decoy for competing for these miRNAs that target PTEN [14], and its overexpression can repress the tumorigenic properties of hepatic carcinoma cells by decoying miR-17, miR-19b and miR-20a [15]. PTENP1 is also found to suppresses GC progression by modulating PTEN [16]. In addition, another pseudogene OCT4pg1 (POU5F1B) is amplified and expressed at a high level in GC, and its amplification confers an aggressive phenotype on GC [17]. To date, only a few pseudogenes are identified in GC and the role of most psedugenes remaining completely unclear. Recently, Han and colleagues detected 9925 pseudogene transcripts in seven cancer types and many of them are tissue and/or cancer-specific, suggesting the potential of pseudogenes as prognostic biomarkers in GC [18].

In this study, we hypothesized that there are still lots of previously unexplored pseudogenes transcripts in human GC. To verify our conjecture, we analyzed the microarray data of GC and normal samples from the Gene Expression Omnibus (GEO), and identified that hundreds of pseudogenes are differentially expressed in GC. Among these psedogenes, DUXAP10 is significantly up-regulated in all datasets. Our previous study has revealed that over-expressed DUXAP10 is associated with poorer prognosis and promotes cell growth in non small cell lung cancer [19]. However, its expression pattern and functional role in human GC remains unclear. In this study, loss- and gain- of function assays were used to investigate the effects of DUXAP10 on GC cells phenotype, and mechanistic investigations were performed to clarify the underlying mechanism of DUXAP10 involved in GC development and progression.

## Methods

### Microarray data analysis

Four public gastric cancer microarray gene profiling datasets (GSE54129, GSE70880, GSE79973, and GSE99416) were downloaded from the Gene Expression Omnibus (GEO). Pseudogene profiling of these microarray datasets was analyzed using the Agilent-038314 CBC *Homo sapiens* lncRNA + mRNA microarray V2.0, Affymetrix Human Genome U133 Plus 2.0 Array, Agilent-045997 Arraystar human lncRNA microarray V3 platforms. The TCGA cancer tissue and normal tissue samples RNA sequencing data was obtained from http://ibl.mdanderson.org/tanric/_design/basic/download.html. All these microarray data and TCGA RNA sequencing data were preprocessed by using R software and packages.

### Clinical specimens and cell lines

Gastric cancer specimen and the corresponding adjacent noncancerous tissues were obtained from Jiangsu Province Hospital between 2010 and 2011 with informed consent. The patients were diagnosed with gastric cancer based on histopathological evaluation, and no local or systemic treatment was conducted before surgery. The protocols used in the study were approved by the Research Ethics Committee of Nanjing Medical University. BGC823, SGC7901, MGC803, AGS, HGC27, MKN45 gastric cancer cell lines and a normal gastric epithelium cell line (GES-1) were purchased from the Shanghai Cell Bank of Chinese Academy of Sciences (Shanghai, China). BGC823, MGC803 and MKN45 cells were cultured in RPMI 1640; SGC7901, AGS and HGC27 were cultured in DMEM medium with 10% fetal bovine serum (FBS) (Invitrogen, Carlsbad, CA, USA). All cell lines were characterized by DNA fingerprinting analysis using short tandem repeat markers at the bank.

### RNA extraction and qPCR assays

Total RNA from specimens and cells was isolated with TRIzol reagent (Invitrogen) according to the manufacturer’s instructions. One Microgram RNA was reverse transcribed in a final volume of 20 μl using random primers under standard conditions for the PrimeScript RT reagent Kit (TaKaRa, Dalian, China). SYBR Premix Ex Taq (TaKaRa, Dalian, China) was used for Quantitative real-time PCR (qPCR) assays, which was carried out on Applied Biosystems 7500 Real-Time PCR System (Applied Biosystems). The specific primers used are presented in Additional file 1: Table S1. The qPCR results were analyzed and expressed relative to threshold cycle (CT) values, and then converted to fold changes.

### Cell transfection

Human DUXAP10 cDNA and short-hairpin RNA directed against DUXAP10 was inserted into the pCDNA3.1 and pLKO.1-TRC vector. Plasmid vectors (pCDNA3.1-DUXAP10, sh-DUXAP10 and empty vectors) for transfection were prepared using DNA Midiprep or Midiprep kits (Qiagen, Hilden, Germany), and transfected into GC cells. The si-DUXAP10, si-EZH2, si-LSD1 or negative control siRNAs were used to knockdown their expression, and all siRNA and shRNA sequence were shown in Additional file 1: Table S1. GC cells were grown in 6-well plates and transfected by Lipofectamine 2000 (Invitrogen) according to the manufacturer’s instructions. At 48 h post-transfection, cells were harvested for qPCR or western blot analysis.

### Cell proliferation, migration and invasion assays

Cell proliferation ability was examined using a Cell Proliferation Reagent Kit I (MTT) (Roche Applied Science) and EdU assay kit (Life Technologies Corporation Carlsbad, CA, USA). Colony formation assays were performed to monitor GC cells cloning capability. FACS analysis for cell cycle progression was done using CycleTEST™ PLUS DNA Reagent Kit (BD Biosciences) after 48-h transfection according to the manufacturer’s protocol. For the migration and invasion assays, cells were placed into the upper chamber of an insert with Matrigel or not (8-μm pore size; Millipore), medium containing 10% FBS was added to the lower chamber. After incubation for 24 h, the cells remaining on the upper membrane were removed with cotton wool, while cells that had migrated or invaded through the membrane were stained with 0.1% crystal violet. Experiments were independently repeated three times.

### In vivo tumor formation assay

Four weeks female athymic BALB/c nude mice were maintained under specific pathogen-free conditions and manipulated according to protocols approved by the Shanghai Medical Experimental Animal Care Commission. sh-DUXAP10 or empty vector stably transfected BGC823 cells were harvested. For tumor formation assay, 10^7^ cells was subcutaneously injected into a single side of each mouse. Tumor growth was examined every 3 days, and tumor volumes were calculated using the eq. V = 0.5 × D × d2 (V, volume; D, longitudinal diameter; d, latitudinal diameter). This study was carried out in strict accordance with the recommendations in the Guide for the Care and Use of Laboratory Animals of the National Institutes of Health. The protocol was approved by the Committee on the Ethics of Animal Experiments of the Nanjing medical University.

### RNA immunoprecipitation

RNA immunoprecipitation was used to investigate whether DUXAP10 could interact or bind with the potential RNA binding proteins (EZH2, SUZ12, LSD1, DNMT1 and HuR) in GC cells. We used the EZMagna RIP kit (Millipore, Billerica, MA, USA) following the manufacturer’s protocol. BGC-823 and SGC-7901 cells were lysed in complete RIP lysis buffer, and the extract was incubated with magnetic beads conjugated with antibodies that recognized EZH2, SUZ12, DNMT1, LSD1 or control IgG (millipore) for 6 h at 4 °C. Then, the beads were washed and incubated with Proteinase K to remove proteins. Finally, purified RNA was subjected to qRT-PCR analysis to demonstrate the presence of DXAP10 using specific primers.

### RNA pull-down assays

DUXAP10 transcripts were transcribed using T7 RNA polymerase (Ambio life) in vitro, then by using the RNeasy Plus Mini Kit (Qiagen) and treated with DNase I (Qiagen). Purified RNAs were biotin-labeled with the Biotin RNA Labeling Mix (Ambio life). Positive control, negative control and Biotinylated RNAs were incubated with BGC823 cell lysates. Then, magnetic beads were added to each binding reaction, and incubated at room temperature. Finally, the beads were washed, and the eluted proteins were detected by western blot analysis.

### Chromatin immunoprecipitation

BGC-823 and SGC-7901 cells were treated with formaldehyde and incubated for 10 mins to generate DNA-protein cross-links. Cell lysates were then sonicated to generate chromatin fragments of 200-300 bp and immunoprecipitated with EZH2, SU12, LSD1 and H3K27me3 and H3K4me2-specific antibodies (Millipore) or IgG as control. Precipitated chromatin DNA was recovered and analyzed by qRT-PCR.

### Fluorescence in situ hybridization and subcellular fractionation location

GC cells were fixed in 4% formaldehyde for 15 min followed, then washed with PBS. Next, the fixed cells were treated with pepsin (1% in 10 mM HCl) and dehydration through 70%, 90% and 100% ethanol. The air-dried cells were further incubated with 40 nM FISH probe in hybridization buffer (100 mg/ml dextran sulfate, 10% formamide in 2 × SSC) at 80 °C for 2 min. The hybridization was performed at 55 °C for 2 h and the slide was washed followed by dehydration. Finally, the air-dried slide was mounted with Prolong Gold Antifade Reagent with DAPI for detection. RNA FISH probe were designed and synthesized by Bogu Co., Ltd. (Shanghai, China). The separation of nuclear and cytosolic fractions was performed using the PARIS Kit (Life Technologies) according to the manufacturer’s instructions.

### Western blot assay and antibodies

BGC823 and SGC7901 cells were lysed with RIPA extraction reagent (Beyotime, Beijing, China) supplemented with a protease inhibitor cocktail (Roche, CA, USA) and phenylmethylsulfonyl fluoride (Roche). Forty microgram protein were separated by 10% SDS-polyacrylamide gel electrophoresis (SDS-PAGE), transferred to 0.22 μm pvdf membranes (Millipore) and incubated with specific antibodies. ECL chromogenic substrate was used to were quantified by densitometry (Quantity One software; Bio-Rad). β-actin antibody was used as control.

### Statistical analysis

The Students t test (2 tailed), one-way ANOVA, and Mann-Whitney U test were conducted to analyze the in vitro and in vivo data by SPSS 17.0 software. *P* values less than 0.05 were considered significant.

## Results

### Identification of differentially expressed pseudogenes in human gastric cancer

**Fig. 1:**
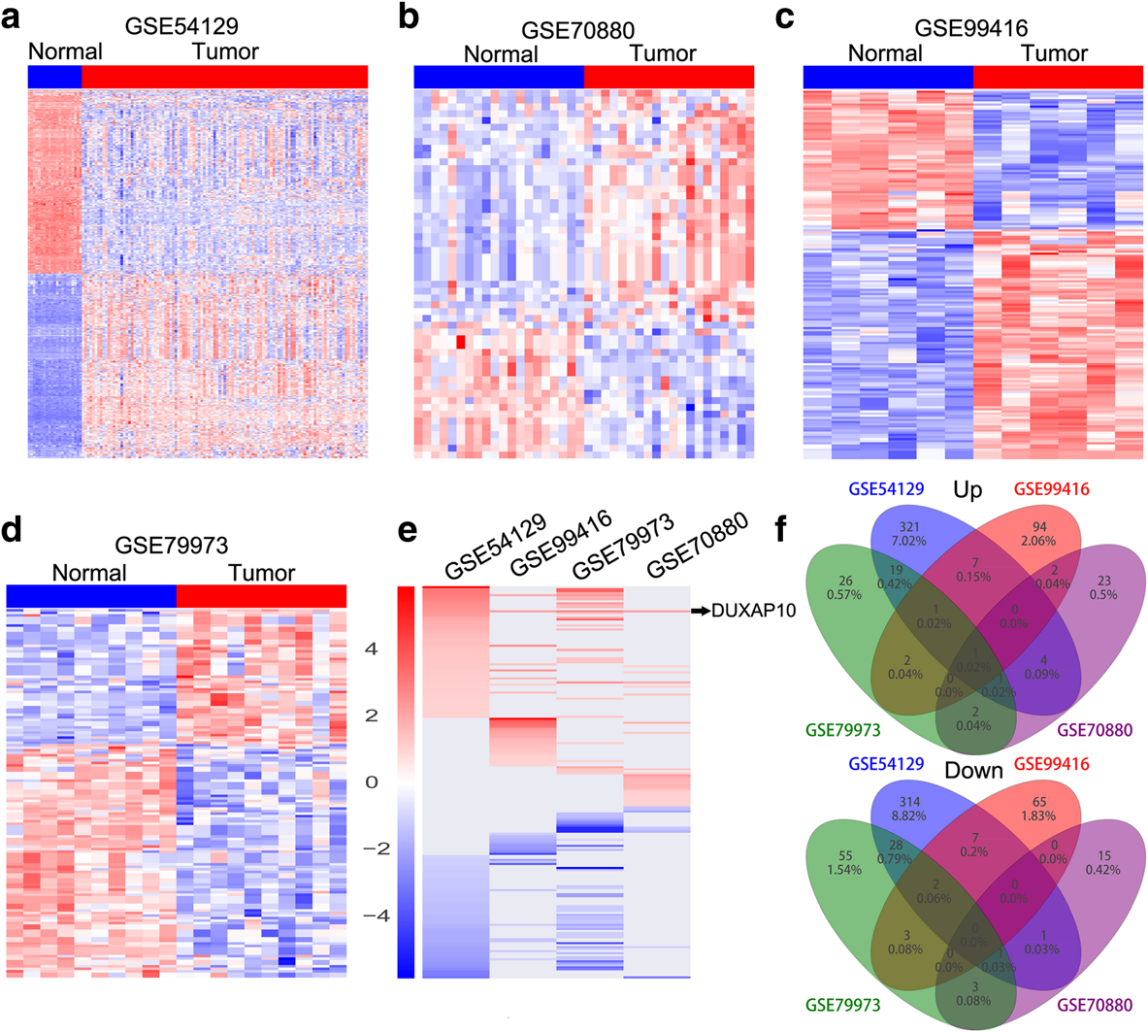
Identification of pseudogenes profiles in human gastric cancer. **a**-**d** The heatmaps were drawn to show the differentially expressed pseudogenes profiles in human gastric cancer and normal tissues using microarray data GSE54129, GSE70880, GSE79973, and GSE99416 from GEO. **e** A heatmap was drawn to show the differentially expressed pseudogenes (consistently altered at least two datasets, fold change) in GSE54129, GSE70880, GSE79973, and GSE99416 datasets. **f** Venn diagram of altered pseudogenes profiling in GSE54129, GSE70880, GSE79973, and GSE99416 datasets

To explore the pseudogenes profile in human gastric cancer tissues, we downloaded four microarray gene profiling data (GSE54129, GSE70880, GSE79973, and GSE99416) from GEO. The GSE54129 dataset consists of 21 normal, and 111 GC samples; GSE70880 consists of 20 paired samples; GSE79973 consists of 10 paired samples; GSE99416 consists of 6 paired samples. Re-annotation and analysis of these data revealed that 707 pseudogenes expression were dysregulated in the GSE54129 dataset (354 upregulated and 353 downregulated); 53 pseudogenes were dysregulated in the GSE70880 dataset (33 upregulated and 20 downregulated); 144 pseudogenes expression were dysregulated in the GSE79973 dataset (52 upregulated and 92 downregulated); and 184 pseudogenes were differentially expressed in the GSE99416 dataset (67 upregulated and 77 downregulated) (Fig. 1a-d, and Additional file 2: Table S2). Further Venn analyses showed that 39 pseudogenes were consistently up-regulated and 45 lncRNAs were down-regulated in at least two datasets (Fig. 1e-f). These Findings indicates that hundreds of pseudogenes are deferentially expressed in human gastric cancer, and part of those altered pseudogenes may be novel biomarkers for GC diagnosis.

### DUXAP10 is significantly up-regulated and correlated with poor prognosis in GC

**Fig. 2:**
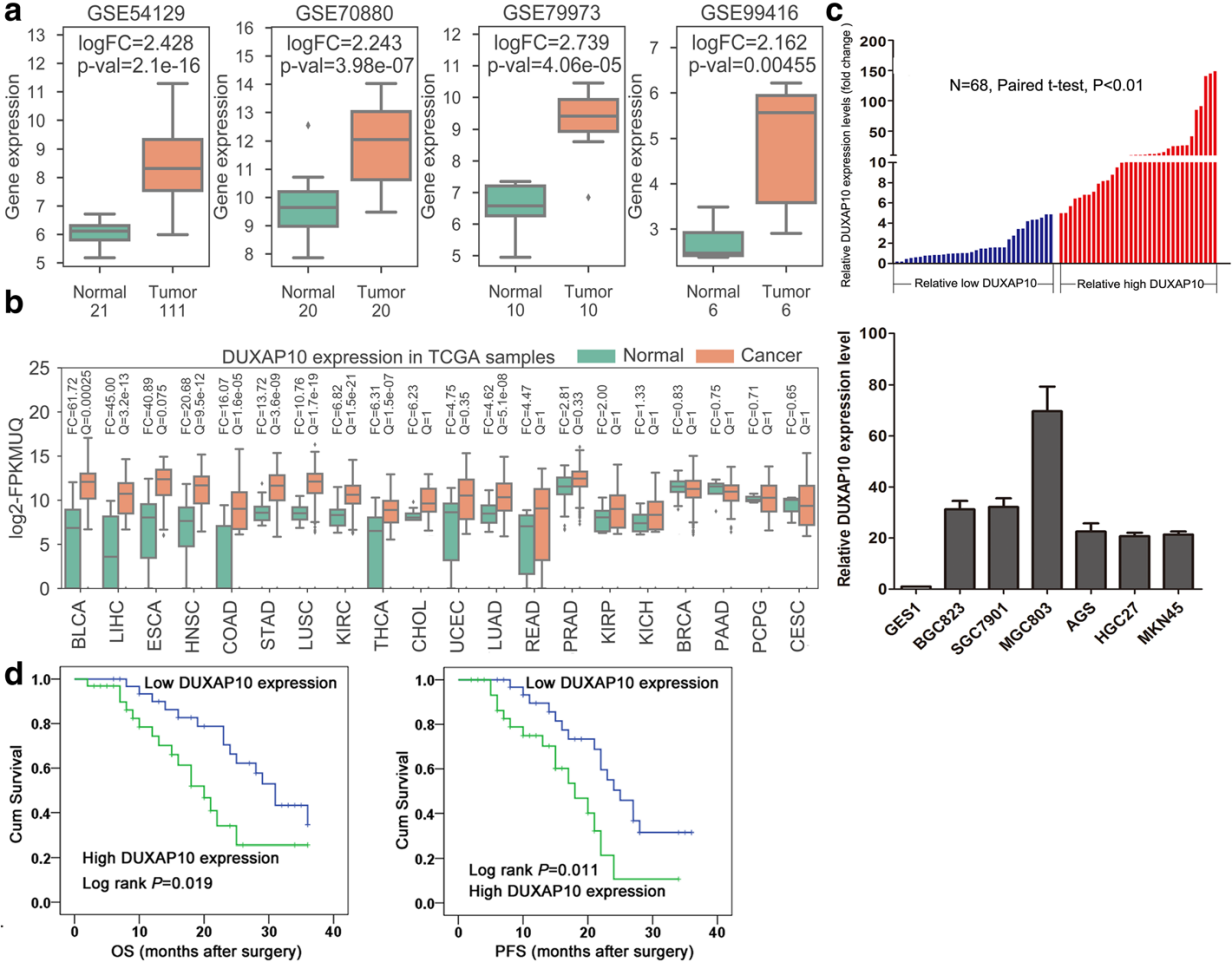
The pseudogene DUXAP10 is over-expressed in gastric cancer tissues and cells. **a** Analysis of the pseudogene DUXAP10 between gastric cancer samples and non-tumor samples in GSE54129, GSE70880, GSE79973, and GSE99416 datasets. **b** Analysis of the pseudogene DUXAP10 between tumor samples and non-tumor samples in 20 cancer types using TCGA RNA sequencing data. **c** The pseudogene DUXAP10 expression level was analyzed by qRT-PCR in gastric cancer samples and adjacent nontumor tissues (*n* = 68), and gastric cancer cell lines. **d** The association of DUXAP10 expression level with gastric cancer patients survival. Kaplan–Meier survival analysis of overall survival and progression free survival time in gastric cancer patients based on DUXAP10 expression

According to the differential expression analysis results of pseudogenes profiles in four microarray datasets, the pseudogene DUXAP10 is the only one that significantly upregulated in all four datasets (Fig. 2a). Interestingly, we further analyzed the expression level of DUXAP10 in 20 types of cancer tissues and normal tissues samples from TCGA and found that DUXAP10 is overexpressed in multiple cancers, such as Colorectal cancer, Thyroid cancer, and Uterine Corpus Endometrial Carcinoma, Pancreas cancer (Fig. 2b). Therefore, we speculate that the pseudogene DUXAP10 may function as an important oncogene in GC development and progression. To further determine the analysis results, we then validated the expression level of DUXAP10 in a total of 68 paired GC tissue and GC cell lines using qPCR. The results showed that DUXAP10 is significantly up-regulated in 50/68 samples (Fold change> 1.5), and 6 gastric cancer cell lines (Fig. 2c).

Next, the patients were divided into two groups: the high DUXAP10 group (*n* = 32, fold-change ≥median), and the low DUXAP10 group (n = 32, fold-change ≤median) to investigate the relationship between DUXAP10 levels and GC patients clinicopathologic feature. The resutls of Pearson’s chi-square tests revealed that increased DUXAP10 expression were correlated with tumor size (*p* = 0.012), advanced pathological stage (*P* = 0.036), and lymph node metastasis (*p* = 0.023). However, DUXAP10 expression was not associated with other factors including gender (*p* = 0.206) and age (*p* = 0.315) in GC (Additional file 2: Table S2). Next, we conducted a Kaplan–Meier survival analysis to explore the correlation between DUXAP10 expression and GC patient prognosis. The results showed that patients with higher DUXAP10 expression levels had a shorter overall survival (OS) and Progression-free survival (PFS) time than those with low DUXAP10 expression (Fig. 2d). Moreover, univariate survival analysis showed that lymph node metastasis, TNM stage and DUXAP10 expression level could be viewed as prognostic factors (Additional file 3: Table S3). Other clinicopathological features including sex and age were not statistically significant prognostic factors. Moreover, multivariate Cox regression analyses showed that expression of DUXAP10 (*p* = 0.034), along with TNM stage (*P* = 0.013), was an independent prognostic factor for gastric cancer patients (Additional file 4: Table S4). These data suggest that over-expressed pseudogene DUXAP10 might play important roles in human gastric cancer tumorigenesis and progression.

### DUXAP10 promotes GC cells proliferation and cell cycle progression in vitro

**Fig. 3:**
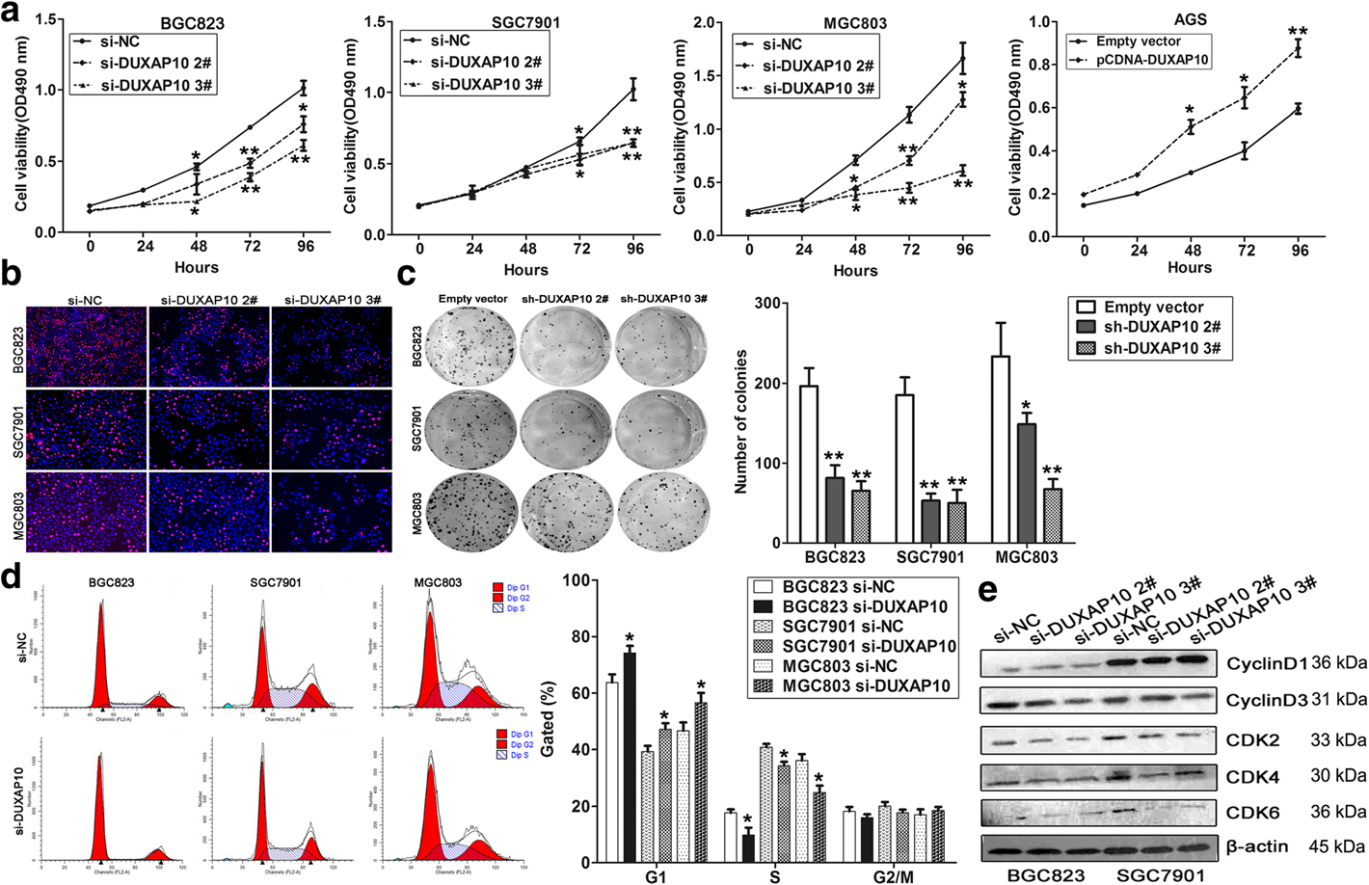
DUXAP10 promotes GC cells growth and cell cycle progression. **a** MTT assays were used to determine the cell viability for si-DUXAP10 or si-NC transfected BGC823, SGC7901 and MGC803 cells, and DUXAP10 vector or empty vector transfected AGS cells. Values represented the mean ± s.d. from three independent experiments. **b** Edu staining analysis showing significant decrease of cell viability in si-DUXAP10 transfected BGC823, SGC7901 and MGC803 cells. **c** Colon formation assays showing significant decrease of cloning viability in si-DUXAP10 transfected GC cells. **d** FACS analysis shows significant increases or decreases of cells in G1or S phase, respectively, in si-DUXAP10 transfected GC cells. **e** Cyclin D1, Cyclin D3, CDK2, CDK4, and CDK6 protein levels were detected by western blot analysis after DUXAP10 knockdown. *P < 0.05, **P < 0.01

To investigate the biological function of DUXAP10 in GC cells, we firstly knocked down DUXAP10 expression in BGC823, SGC7901 and MGC803 cells by transfection with siRNAs or shRNA vector, and up-regulated DUXAP10 by transfected with pCDNA-DUXAP10 vector (Additional file 5: Figure S1a-c). Then, MTT, EdU staining and colony formation assays showed that the growth and colony formation ability of GC cells transfected with si-DUXAP10 were impaired compared with control cells, while DUXAP10 ovexpression promoted AGS cells proliferation (Fig. 3a-c and Additional file 5: Figure S1d-e). To further determine whether the effect of DUXAP10 on proliferation of GC cells reflected cell cycle arrest, cell cycle progression was analyzed by flow cytometry analysis. The results showed that GC cells transfected with si-DUXAP10 had an obvious cell cycle arrest at the G1/G0 phase and a decreased G2/S phase (Fig. 3d). However, the flow cytometry analysis showed that knockdown of DUXAP10 had no effect on GC cells apoptosis (data not shown). Consistent with these data, the levels of well-known cell cycle protein markers including CDK2 and CDK6 were down-regulated in DUXAP10 knockdown cells (Fig. 3e). These data indicate that DUXAP10 could promote the cell cycle progression and proliferation phenotype of GC cells in vitro.

### Down-regulation of DUXAP10 inhibits GC cell tumorigenesis in vivo

**Fig. 4:**
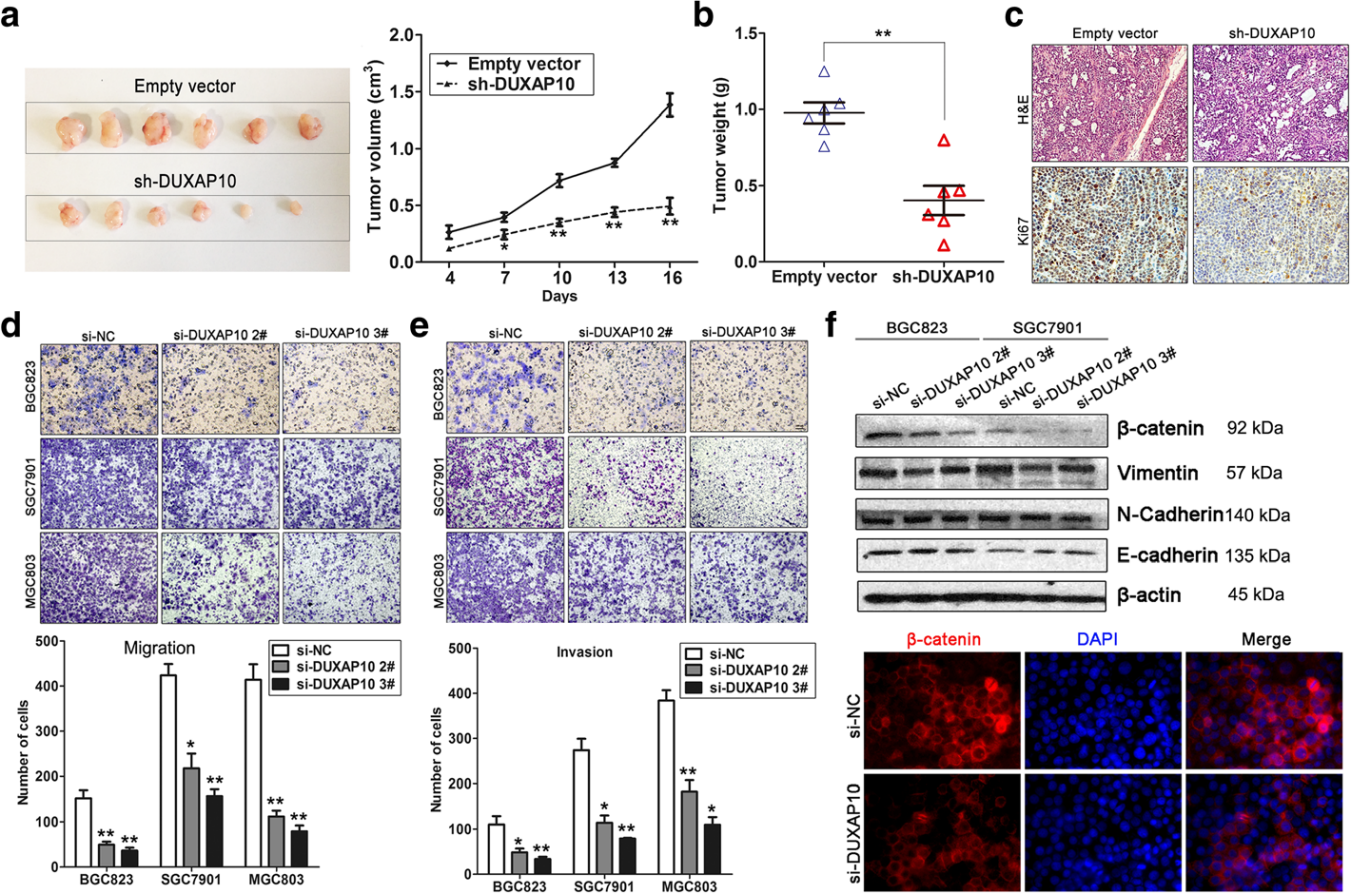
DUXAP10 down-regulation inhibits GC cells tumor growth in vivo, and invasion in vitro. **a** Representative images of tumors formed in nude mice injected subcutaneously with DUXAP10 knockdown BGC823 cells, and the tumor growth curves of DUXAP10 down-regulation and control groups. **b** Tumors induced by DUXAP10 knockdown in BGC823 cells showed markedly lower weight than control cells. **c** Tumors developed from sh-DUXAP10 transfected BGC823 cells showed lower ki67 protein levels than tumors developed by control cells. Up: H & E staining; Down: immunostaining. **d**,**e** Transwell assays were used to investigate the changes in migratory and invasive abilities of DUXAP10 knockdown cells. **f** E-cadherin, N-cadherin, Vimentin and β-catenin protein levels were detected by western blot and Immunofluorescence analysis after DUXAP10 knockdown in BGC823 cells. *P < 0.05, **P < 0.01

To further investigate whether knockdown of DUXAP10 expression could affect tumor growth in vivo, BGC823 cells stably transfected with sh-DUXAP10 or empty vectors were inoculated into male nude mice. Eighteen days after injection, the tumor size in the sh-DUXAP10 group was significantly smaller compared with the control group (Fig. 4a). The tumor weight of sh-DUXAP10 group was also significantly lower than that in the control group(Fig. 4b). Next, qPCR assays determined that DUXAP10 expression levels were down-regulated in tumor tissues collected from sh-DUXAP10 group compared with control group (Additional file 6: Figure S2a). Moreover, immunohistochemistry (IHC) analysis confirmed that the tumors formed from BGC823/sh-DUXAP10 cells displayed lower Ki-67 staining than those formed from the control cells (Fig. 4c). Our results indicated that knockdown of DUXAP10 expression could suppress GC cells tumor growth in vivo.

### Knockdown of DUXAP10 inhibits gastric cancer cells migration and invasion

Cancer cells migration and metastasis is a significant aspect of cancer progression. Then, we investigated the effect of DUXAP10 on GC cells migration and invasion by performing transwell assays. The results revealed that decreased DUXAP10 impeded the gastric cancer cells migration and invasion compared with controls (Fig. 4d-e). Epithelial-Mesenchymal Transition has been found to play important roles in cancer cells invasion and metastasis, and we investigated whether DUXAP10 could affect this process in GC cells by analyzing EMT markers levels after knockdown of DUXAP10. Western blot assays showed that β-catenin was decreased in DUXAP10 knockdown GC cells, while other protein levels had no significant change. In addition, immunofluorescence analysis also showed that β-catenin protein levels were decreased in DUXAP10 knockdown GC cells (Fig. 4f and Additional file 6: Figure S2b).

### DUXAP10 directly binds with PRC2/LSD1/HuR in GC cells

**Fig. 5:**
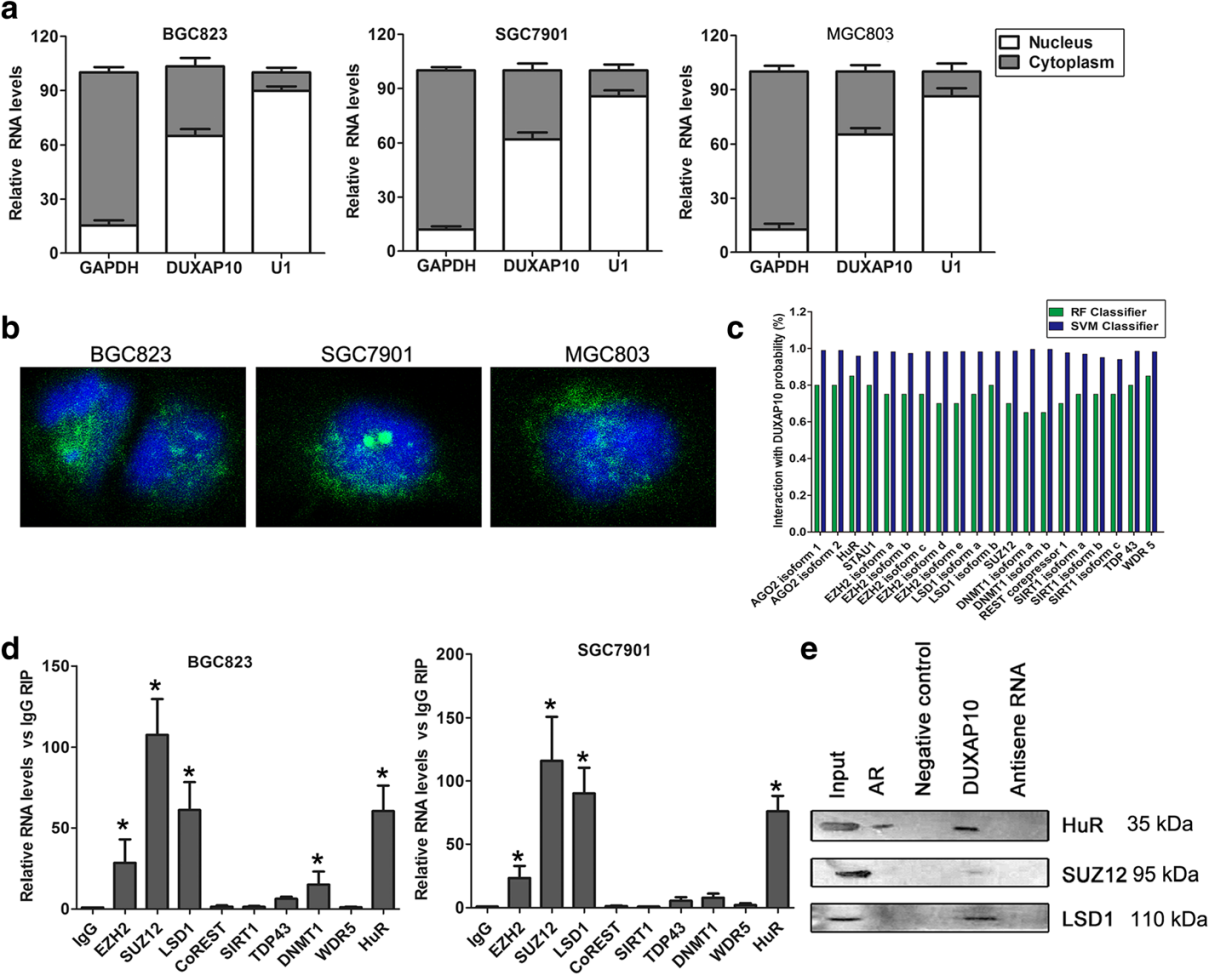
DUXAP10 directly binds with PRC2, LSD1 and HuR in GC cells. **a** DUXAP10 expression levels in cell nucleus or cytoplasm fraction of GC cells were detected by qRT-PCR. U1 was used as a nucleus marker and GAPDH was used as a cytosol marker. **b** FISH was performed to determine the distribution of DUXAP10 in GC cells. **c** RNA–protein interaction prediction (RPISeq) analysis was used to predict the interaction between DUXAP10 and RNA binding proteins (http://pridb.gdcb.iastate.edu/RPISeq/), predictions with probabilities > 0.5 were considered positive. **d** DUXAP10 directly bound to PRC2, LSD1 and HuR in RIP assays using BGC823 and SGC7901 cell extracts. **e** Biotinylated DUXAP10 RNAs or its antisense sequence were incubated with BGC823 cell lysates, targeted with magnetic beads, and associated proteins were resolved electrophoretically. Western blot analysis of the specific association of SUZ12, LSD1, HuR and DUXAP10. AR mRNA interaction with HuR was used as positive control. *P < 0.05, **P < 0.01

Generally, most of the pseudogenes regulate their parental genes expression by acting as endogenous competing RNAs for miRNAs due to their high homology to parental genes. However, we analyzed the sequence of DUXAP10 and its parental gene, and found that DUXAP10 is not homology to its parental gene, suggesting that DUXAP10 may regulate other target genes through different mechanisms. To validate our hypothesis, we firstly analyzed the distribution of DUXAP10 transcript in GC cells. The results showed that DUXAP10 transcript is distributed in both cytoplasm and nucleus, but the ratio of DUXAP10 in nucleus is more higher (Fig. 5a and b). Furthermore, we predicted the interaction probabilities of DUXAP10 and RNA binding protein via RNA-Protein interaction prediction (http://pridb.gdcb.iastate.edu/RPISeq/), and the results showed that DUXAP10 may potentially bind with PRC2, LSD1, WDR, DNMT1, HuR, AGO2 and STAU1 (as the RF or SVM score > 0.5, Fig. 5c). Additionally, RIP assays determined that DUXAP10 only directly binds with PRC2, LSD1 and HuR in GC cells(Fig. 5d). Furthermore, RNA-pulldown confirmed that DUXAP10 could bind with SUZ12, LSD1 and HuR in GC cells (Fig. 5e).

### LATS1 and β-catenin are key downstream mediator of DUXAP10 in GC cells

**Fig. 6:**
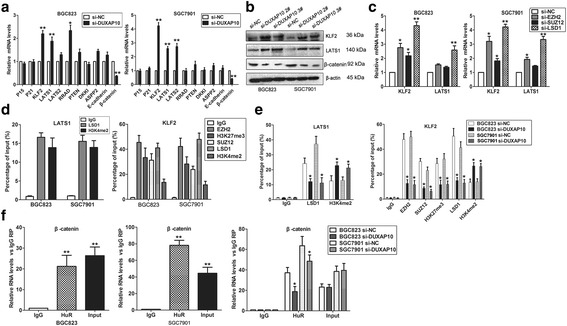
DUXAP10 interacts PRC2/LSD1 and HuR to regulate KLF2, LATS1and β-catenin expression. **a** QPCR was used to examine the levels of potential DUXAP10 targets in GC cells after knockdown of DUXAP10. **b** KLF2, LATS1and β-catenin protein levels were analyzed by western blot in GC cells after knockdown of DUXAP10. **c** KLF2 and LATS1 levels were analyzed by qPCR in GC cells after transfection with EZH2, SUZ12 and LSD1 siRNA. **d**, **e** ChIP shows SUZ12, EZH2, H3K27me3, LSD1 and H3K4me2 occupancy in the LATS1 and KLF2 promoter region, while knockdown of DUXAP10 decreased their binding ability. **f** RIP assays show the interaction between HuR and β-catenin mRNA in GC cells, and knockdown of DUXAP10 impaired their interaction ability. *P < 0.05, **P < 0.01

To further explore the underlying target genes of DUXAP10 in GC cells, we analyzed previously published gene expression profile downstream of LSD1 in breast cancer cells and other known LSD1 and PRC2 targets. The qPCR assay was performed to examine the expression of these genes after knockdown of DUXAP10 in BGC823 and SGC7901 cells. The results showed that DUXAP10 down-regulation increased the expression of KLF2 and LATS1, and decreased β-catenin expression in both BGC823 and SGC7901 cells (Fig. 6a). Similarly, western blot analysis showed the same results (Fig. 6b). To determine whether DUXAP10 repressed KLF2 and LATS1 expression via interacting with PRC2 or LSD1 in GC cells, we evaluated their expression after knockdown of EZH2, SUZ12, and LSD1 in GC cells. Interestingly, either knockdown of EZH2, SUZ12 or LSD1 upregulated KLF2 while only knockdown of LSD1 increased LATS1 expression (Fig. 6c). To further determine whether PRC2 or LSD1 could directly bind to the promoter region of KLF2 and LATS1, we designed four pairs of primers across 2000 bp of their promoter regions. ChIP assays confirmed that PRC2 and LSD1 could bind to the KLF2 promoter region, and LSD1 could bind to the LAST1 promoter region(Fig. 6d). Moreover, knockdown of DUXAP10 reduced their binding to KLF2 or LATS1 promoter regions (Fig. 6e).

Previous studies reveal that RNA binding protein HuR could stabilize the β-catenin mRNA and increase its protein levels in HCC cells which is mediated by lncRNA UFC1 [20]. Here, we also found DUXAP10 could bind with HuR in GC cells and knockdown of its expression decrease β-catenin protein level. More important, RIP assays showed that HuR could also directly bind with β-catenin mRNA in GC cells, and knockdown of DUXAP10 decreased their binding ability (Fig. 6f). These results suggest that DUXAP10 may also recruit HuR to stabilize β-catenin mRNA, resulted in increased β-catenin protein level and promoting GC cells metastasis.

### Silencing of LATS1 is partly involved in the oncogenic function of DUXAP10

**Fig. 7:**
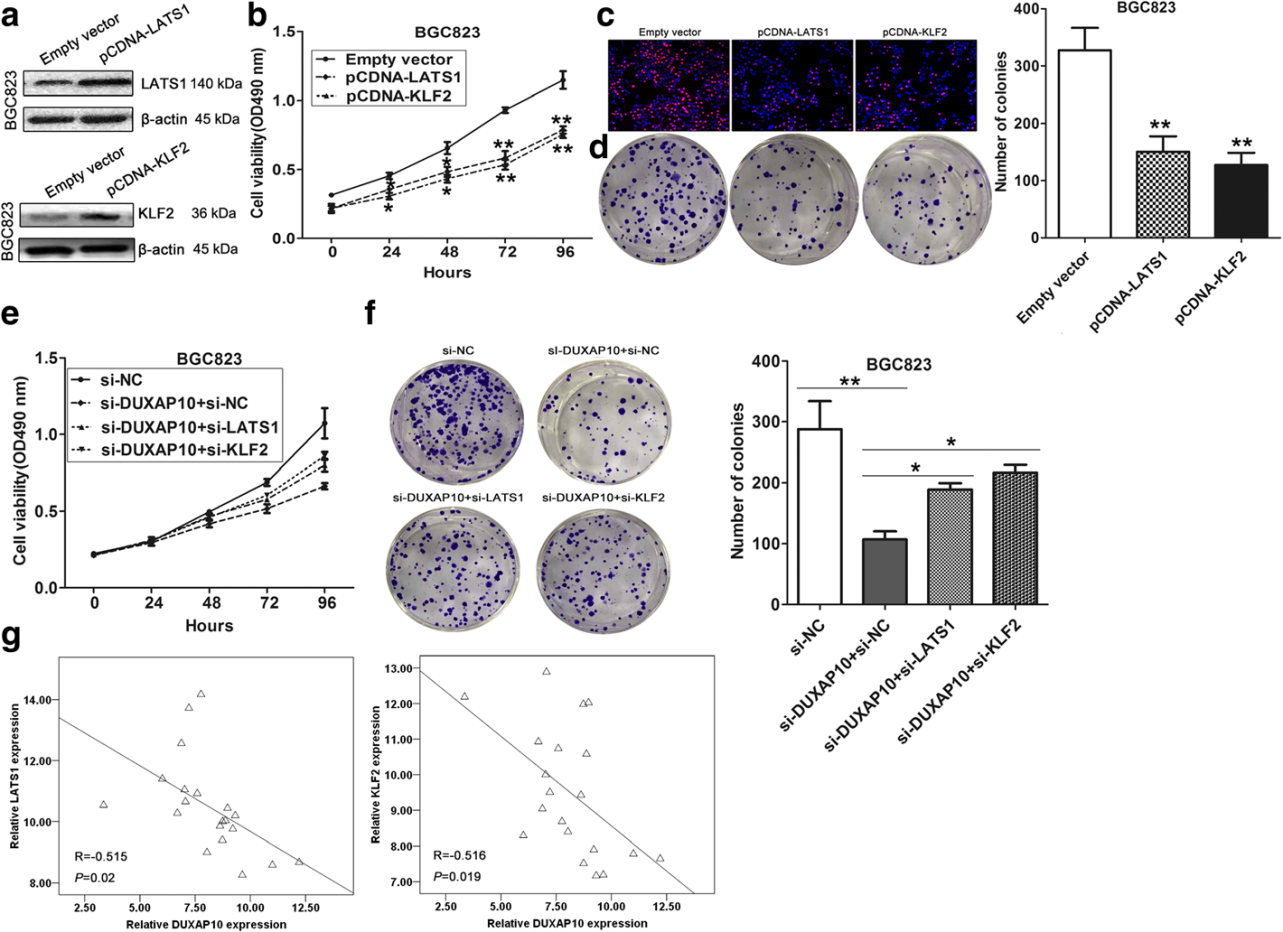
DUXAP10 promotes GC cell proliferation partly via regulating LATS1 and KLF2. **a** KLF2 and LATS1 protein levels were detected by western blot in BGC823 cells transfected with KLF2 or LATS1 vector. **b** MTT assays were used to determine the cell viability for LATS1 and KLF2 vector or empty vector transfected BGC823 and SGC7901 cells. **c**,**d** Edu staining and colony formation assays were used to determine the cell viability for LATS1, KLF2 vector or empty vector transfected cells. **e**,**f** MTT and colony formation assays showed that cell proliferation was partly rescued by KLF2 and LATS1 knockdown in DUXAP10 siRNA transfected cells. **g** The correlation between DUXAP10 and KLF2, or LATS1 expression was detected in 20 pairs of GC and corresponding noncancerous tissues by qRT-PCR

To further investigate whether LATS1 and KLF2 are involved in the DUXAP10 induced promotion of GC cells proliferation, we performed gain-of-function assays. The western blot assays confirmed that LATS1 and KLF2 expression was significantly up-regulated in BGC823 cells transfected with pCDNA-LATS1 and pCDNA-KLF2 compared with control cells (Fig. 7a). MTT, EdU and colon formation assays demonstrated that the GC cell proliferation was inhibited upon overexpression of KLF2 and LATS1 (Fig. 7b-d). Moreover, we conducted rescue assays to determine whether KLF2 and LATS1 involved in DUXAP10 contributions to GC cell proliferation. BGC823 cells were co-transfected with si-DUXAP10, si-KLF2 or si-LATS1. The MTT and colony formation assays showed that si-KLF2 or si-LATS1 transfection could partly rescue si-DUXAP10 decreased GC cells growth (Fig. 7e and f). These findings indicate that DUXAP10 exerting oncogenic effects in GC cells may partly through repressing KLF2 and LATS1 expression. Finally, we analyzed the correlation between DUXAP10, LATS1 and KLF2 expression in 20 pair GC tissues, and found that there was a significantly negative correlation between DUXAP10 and KLF2 or LATS1 (Fig. 7g).

## Discussion

In recent years, completion of the human genome sequencing and DNA cloning projects reveals that majority of the human genome is transcribed, but most of these transcripts represent non protein-coding genes [21, 22]. Over past decade, microRNAs and lncRNAs aberrant expression are found to be implicated in the development and progression of cancers [8, 23]. Recently, a handful of investigations have highlighted the involvement of another important sub-class of non-coding genes-pseudogenes during pathogenesis of diseases, especially in cancer [13, 24–26]. Although pseudogenes have been considered as non-functional relics littering the genome for long time, it is clearly that thousands of pseudogenes are transcribed as sense transcripts. Importantly, few of them were found to regulate cancer cells phenotype through acting as sponges/decoys for miRNAs and proteins or pseudogene asRNAs-mediated regulation. For example, pseudogene-OCT4-pg4 is abnormally activated in hepatocellular carcinoma (HCC) and its high level is significantly correlated with poor prognosis of HCC patients. Mechanistic investigation revealed that OCT4-pg4 functions as a ceRNA to protect OCT4 transcript from being inhibited by miR-145, thus promoting HCC cells growth and tumorigenicity [27]. In addition, Ye et al. reported an actively transcribed VEGFR1/FLT1 pseudogene (FLT1P1) that is transcribed bidirectionally in human colorectal cancer cells, and knockdown of its expression markedly inhibited CRC cells proliferation and tumor growth by inhibiting the VEGFR1 and VEGF-A expression through interacting with miR-520a in CRC cells [28]. These findings indicate that pseudogenes may be an important missing piece of the molecular regulation network puzzle of cancer.

To date, only a few intriguing reports reveal the involvement and underlying mechanisms of pseudogenes in human GC, while the biological function of the great majority of annotated pseudogenes currently remains unknown. In this study, we performed comprehensive analysis of pseudogenes profiles in human gastric cancer and identified hundreds of pseudogenes were differentially expressed. Subsequently, one of these altered pseudogenes termed DUXAP10 is frequently overexpressed in multiple cancers including liver hepatocellular carcinoma, bladder cancer, esophageal Cancer, and gastric cancer tissues. Moreover, higher DUXAP10 expression is significantly associated with gastric cancer patients poorer prognosis and shorter survival time. The investigation of biologic consequences of DUXAP10 in regulating GC cell phenotype showed that knockdown of DUXAP10 not only impaired GC cell proliferation and in vivo growth, but also inhibited GC cells invasive activity. These findings indicate that DUXAP10 may be an important oncogenic pseudogene in human cancers.

As homology to their parental genes, many pseudogenes involved in the regulation of multiple biologic processes though regulating their parental genes expression by acting as ceRNAs. In addition, a few of studies showed that the pseudogene or its antisense RNA can also recruitment of regulatory proteins to complementary sites to modulate chromatin remodeling and transcription or competition for RNA-binding proteins [29, 30]. However, whether pseudogene could regulate other genes not their parental genes through different mechanisms in cancers is not clear. Here, we reported that DUXAP10 could not only repress tumor suppressors LATS1 and KLF2 transcription through epigenetic modification by interacting with PRC2 and LSD1, also can regulate β-catenin mRNA stability by recruiting RNA binding protein HuR at post-transcriptional level in GC cells. Our findings provide evidence that pseudogenes transcript could also regulate other genes not their parental genes through interacting with histone protein modification enzymes or other RNA binding protein.

KLF2 is an member of KLF family that with Cys2/His2 zinc-finger domains [31]. There is evidence shows that its expression is diminished in multiple cancers and possesses tumor-suppressor features [32]. Our previous study shows that SUZ12 could repress KLF2 expression in GC cells. In this study, we also found that KLF2 can function as tumor suppressor and its’ expression could be suppressed by DUXAP10 through recruiting PRC2 and LSD1 to its promoter region in GC cells. Meanwhile, DUXAP10 also silenced LATS1 in GC cells through recruiting LSD1 to its promoter region. LATS1 is a member of large tumor suppressor (LATS), which is a Ser/Thr kinase belonging to the Ndr/LATS subfamily of AGC (protein kinase A/PKG/PKC) kinases [33]. Recent studies show that LATS1 is a central regulator of an emerging tumor suppressor pathway termed the Hippo-LATS/Warts pathway that suppresses tumor growth [34], and we also found that LATS1 could suppress GC cells proliferation. Interestingly, DUXAP10 can also exert oncogenic function by increasing β-catenin protein levels through post-transcription regulation in cytoplasm, while increased β-catenin protein leads to promotion of GC cells invasion and metastasis.

## Conclusion

In conclusion, our study reveals that pseudogene DUXAP10 expression is significantly up-regulated in GC tissues and cells, indicating that its overexpression may be a negative prognostic factor for GC patients. Knockdown of DUXAP10 exerts tumor-suppressive functions through reducing cell proliferation, growth and invasion. Our findings may further the understanding of GC pathogenesis, and facilitate the development of pseudogene-directed diagnostics and therapeutics against this disease. However, whether DUXAP10 could regulate other possible targets and the mechanism that underlie regulatory behaviors are not investigated in this study, which needs to be further investigated.

## Additional files

Additional file 1: Table S1. Primer, siRNA and shRNA sequence, and antibodies information. (XLS 55 kb)

Additional file 2: Table S2. Pseudogenes profiling in gastric cancer from four GEO datasets. (XLSX 61 kb)

Additional file 3: Table S3. Correlation between DUXAP10 expression and clinicopathological characteristics of gastric cancer patients (*n* = 64). (DOC 38 kb)

Additional file 4: Table S4. Univariate and multivariate analysis of over-survival in gastric cancer patients (*n* = 64). (DOC 38 kb)

Additional file 5: Figure S1. (a, b) Analysis of the pseudogene DUXAP10 expression levels in BGC823, SGC7901, and MGC803 cells after transfection with DUXAP10 siRNAs or shRNAs by qPCR. (c) Analysis of DUXAP10 expression levels in AGS cells after transfection with DUXAP10 over-expression vector. (d, e) EdU incorporation and colony formation assays were performed to evaluate the effect of DUXAP10 over-expression on AGS cells proliferation. *P < 0.05 and **P < 0.01 (TIFF 9825 kb)

Additional file 6: Figure S2. (a) Analysis of DUXAP10 expression levels in tumor tissues collected from sh-DUXAP10 group and control group mice by qPCR. (b) Statistical analysis of E-cadherin, N-cadherin, Vimentin and β-catenin protein levels in DUXAP10 or negative control siRNAs transfected cells. *P < 0.05 and **P < 0.01 (TIFF 4831 kb)

## Abbreviations

ChIP: Chromatin immunoprecipitation
EZH2: Enhancer of zeste homolog 2
GC: Gastric cancer
H3K27me3: Histone H3 lysine 27 trimethylation
PRC2: Polycomb repressive complex 2
RIP: RNA immunoprecipitation
