# Correction: Over-expression of oncigenic pesudogene DUXAP10 promotes cell proliferation and invasion by regulating LATS1 and β-catenin in gastric cancer

**DOI:** 10.1186/s13046-023-02822-x

**Published:** 2023-09-06

**Authors:** Yongcan Xu, Xiang Yu, Chenchen Wei, Fengqi Nie, Mingde Huang, Ming Sun

**Affiliations:** 1 Department of General Surgery, Huzhou Central Hospital, Huzhou, People’s Republic of China; 2 Department of General Surgery, The Affiliated Yantai Yuhuangding Hospital of Qingdao University, Yantai, People’s Republic of China; 3 Department of Oncology, Second Affiliated Hospital, Nanjing Medical University, Nanjing, People’s Republic of China; 4 Department of Oncology, First Affiliated Hospital, Nanjing Medical University, Nanjing, People’s Republic of China; 5 Department of Oncology, Huai’an First People’s Hospital, Nanjing Medical University, Huai’an, People’s Republic of China; 6 Department of Bioinformatics and computational biology, UT MD Anderson Cancer Center, 1400 Pressler Street, Unit 1410, Houston, TX 77030 USA

**Correction:** *J Exp Clin Cancer Res* **37, 13 (2018)**

10.1186/s13046-018-0684-8

Following publication of the original article [1], an error was identified in Figs. 3b and 4d/e, and Fig. 7c.

The corrected figures are given below. The corrections do not affect the conclusions of the article.

**Fig. 3:**
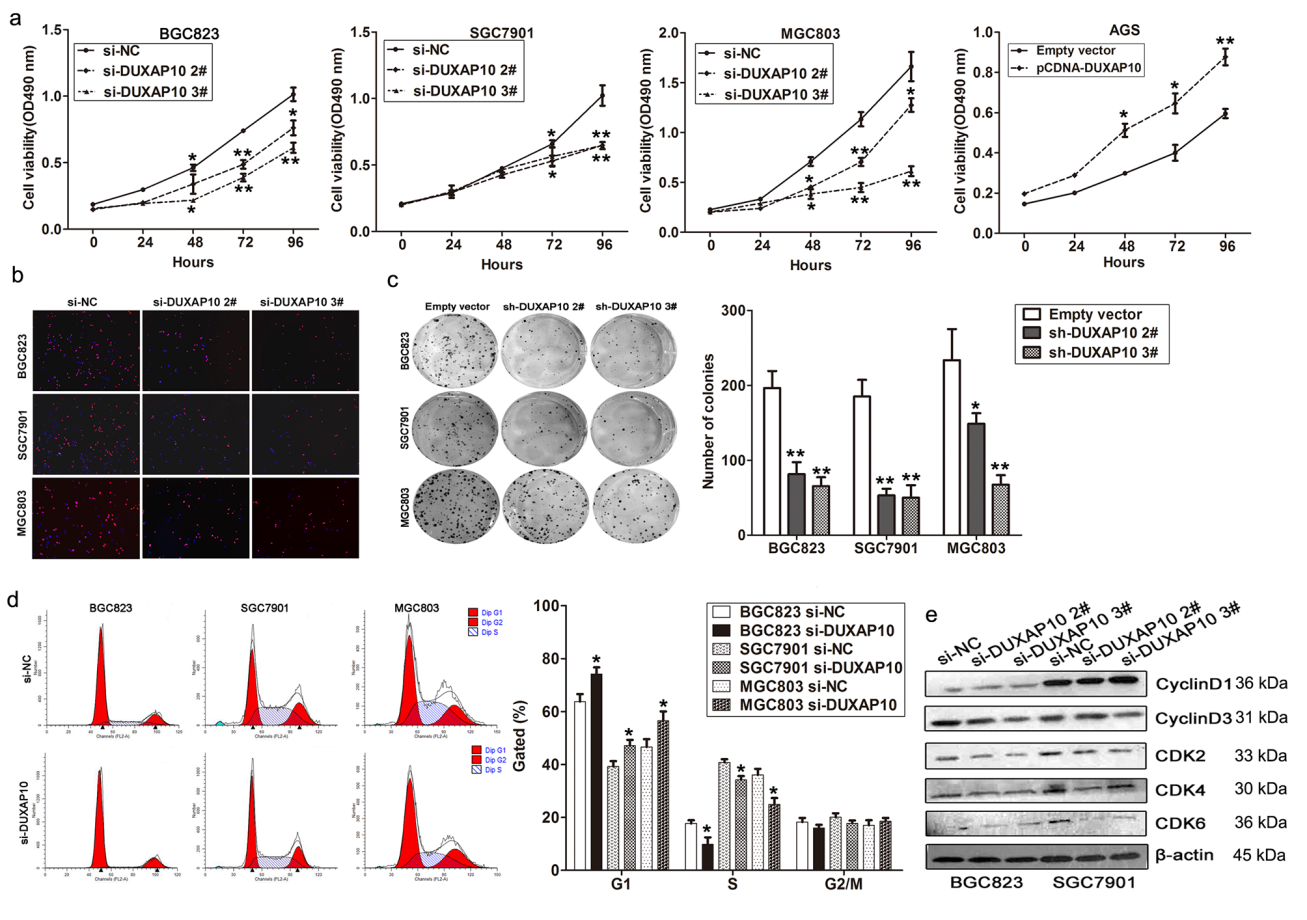
DUXAP10 promotes GC cells growth and cell cycle progression. **a** MTT assays were used to determine the cell viability for si-DUXAP10 or si-NC transfected BGC823, SGC7901 and MGC803 cells, and DUXAP10 vector or empty vector transfected AGS cells. Values represented the mean ± s.d. from three independent experiments. **b** Edu staining analysis showing significant decrease of cell viability in si-DUXAP10 transfected BGC823, SGC7901 and MGC803 cells. **c** Colon formation assays showing significant decrease of cloning viability in si-DUXAP10 transfected GC cells. **d** FACS analysis shows significant increases or decreases of cells in G1or S phase, respectively, in si-DUXAP10 transfected GC cells. **e** Cyclin D1, Cyclin D3, CDK2, CDK4, and CDK6 protein levels were detected by western blot analysis after DUXAP10 knockdown. *P < 0.05, **P < 0.01

**Fig. 4:**
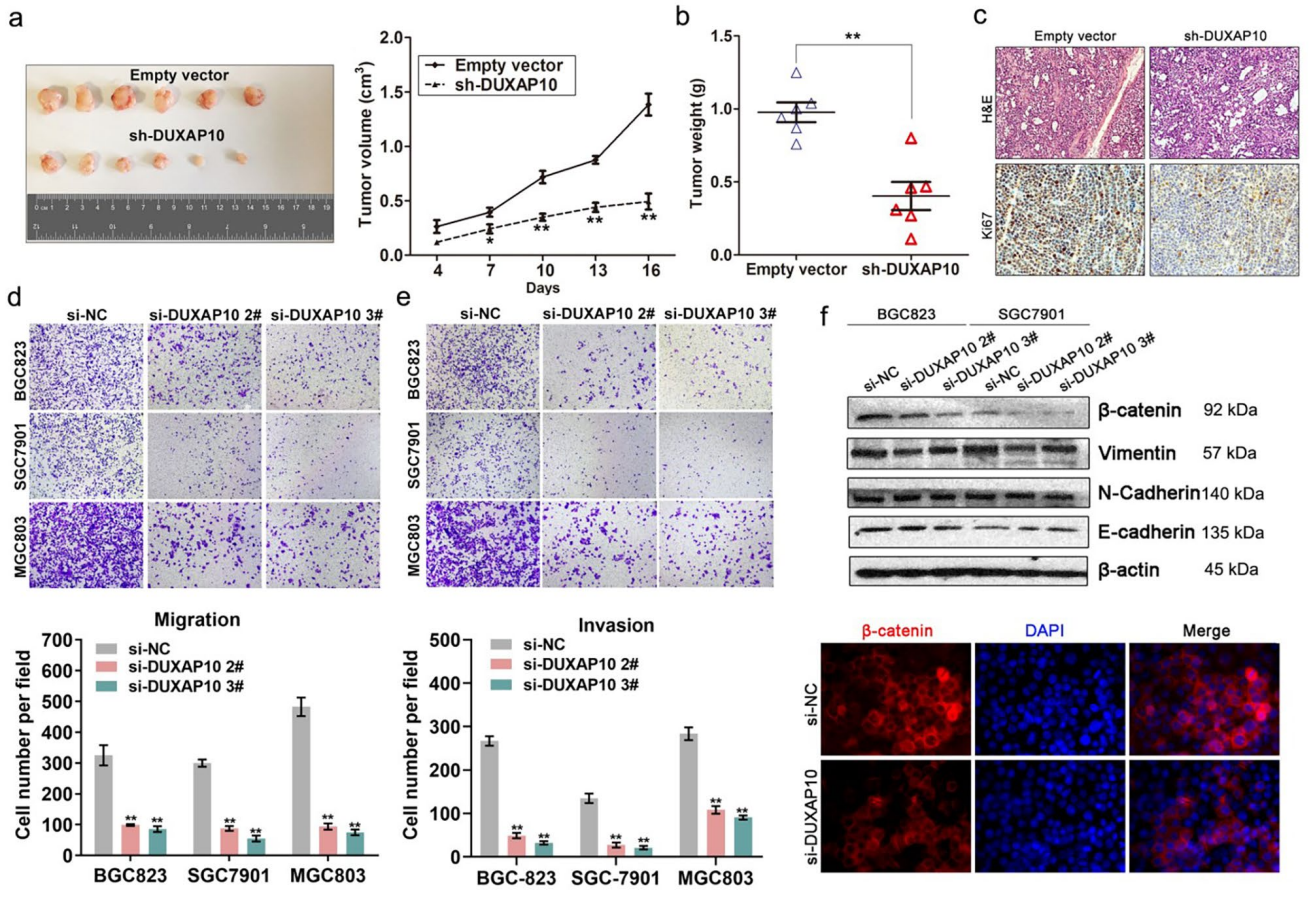
DUXAP10 down-regulation inhibits GC cells tumor growth in vivo, and invasion in vitro. **a** Representative images of tumors formed in nude mice injected subcutaneously with DUXAP10 knockdown BGC823 cells, and the tumor growth curves of DUXAP10 down-regulation and control groups. **b** Tumors induced by DUXAP10 knockdown in BGC823 cells showed markedly lower weight than control cells. **c** Tumors developed from sh-DUXAP10 transfected BGC823 cells showed lower ki67 protein levels than tumors developed by control cells. Up: H & E staining; Down: immunostaining. **d,e** Transwell assays were used to investigate the changes in migratory and invasive abilities of DUXAP10 knockdown cells. **f** E-cadherin, N-cadherin, Vimentin and β-catenin protein levels were detected by western blot and Immunofluorescence analysis after DUXAP10 knockdown in BGC823 cells. *P < 0.05, **P < 0.01

**Fig. 7:**
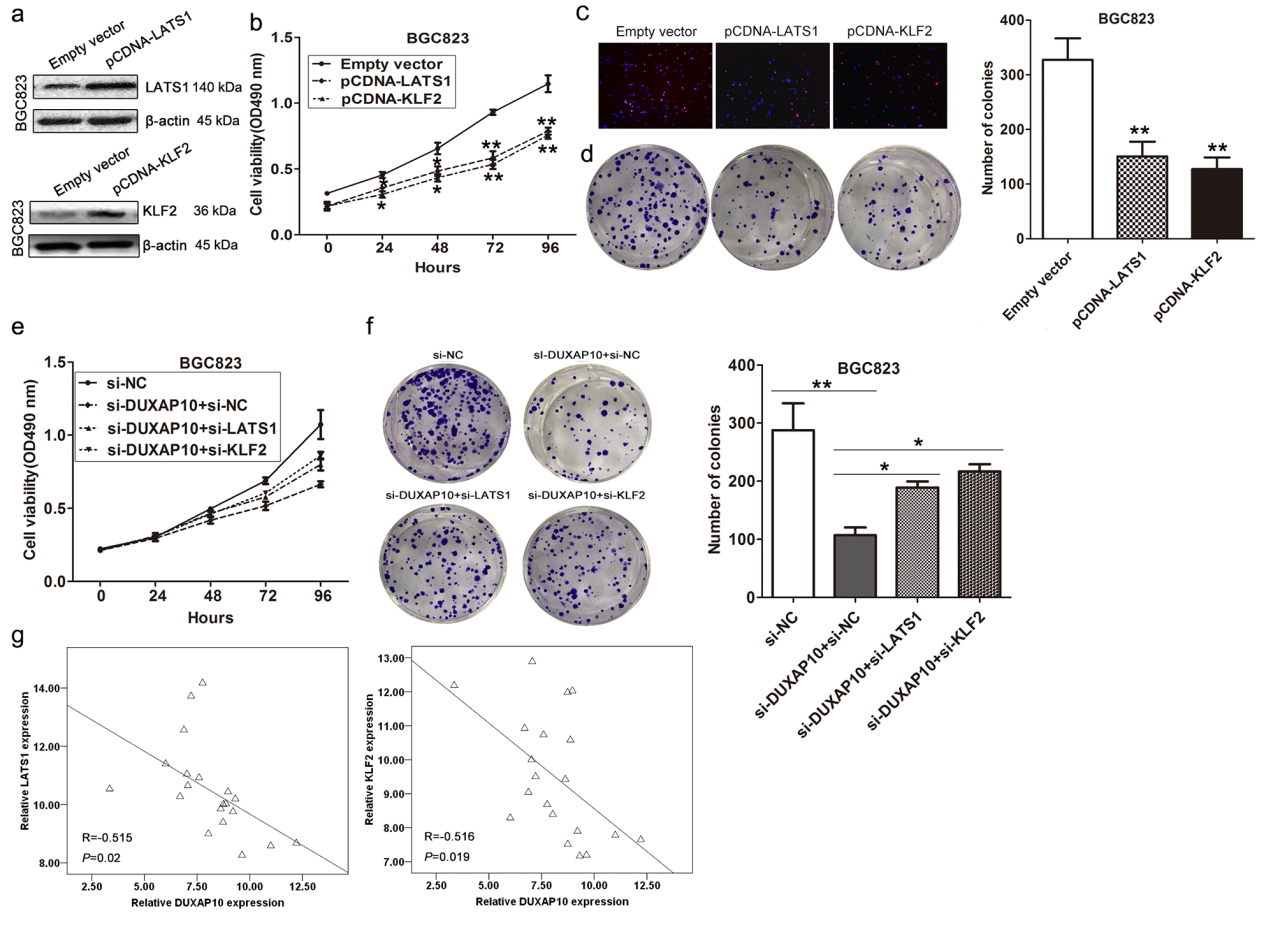
DUXAP10 promotes GC cell proliferation partly via regulating LATS1 and KLF2. **a** KLF2 and LATS1 protein levels were detected by western blot in BGC823 cells transfected with KLF2 or LATS1 vector. **b** MTT assays were used to determine the cell viability for LATS1 and KLF2 vector or empty vector transfected BGC823 and SGC7901 cells. **c,d** Edu staining and colony formation assays were used to determine the cell viability for LATS1, KLF2 vector or empty vector transfected cells. **e,f** MTT and colony formation assays showed that cell proliferation was partly rescued by KLF2 and LATS1 knockdown in DUXAP10 siRNA transfected cells. **g** The correlation between DUXAP10 and KLF2, or LATS1 expression was detected in 20 pairs of GC and corresponding noncancerous tissues by qRT-PCR
