# Supplementary material for: Over-expression of oncigenic pesudogene DUXAP10 promotes cell proliferation and invasion by regulating LATS1 and β-catenin in gastric cancer
Source: J Exp Clin Cancer Res. 2018 Jan 27;37:13. doi: 10.1186/s13046-018-0684-8 (PMC5787324; doi:10.1186/s13046-018-0684-8)
Supplement: Supplementary file 3 — Correlation between DUXAP10 expression and clinicopathological characteristics of gastric cancer patients (n = 64). (DOC 38 kb) [file 13046_2018_684_MOESM3_ESM.doc]

**Table S3** Correlation between DUXAP10 expression and clinicopathological characteristics of gastric cancer patients (n=64).

| **Characteristics** |  | **DUXAP10** | | **P value** |
| --- | --- | --- | --- | --- |
|  |  | **Low** | **High** |  |
| **Age** | <50 | 12 | 17 | **0.315** |
|  | >50 | 20 | 15 |  |
| **Gender** | Male | 16 | 21 | **0.206** |
|  | Fmale | 16 | 11 |  |
| **location** | Distal | 14 | 11 | **0.296** |
|  | Middle | 10 | 26 |  |
|  | Proximal | 8 | 5 |  |
| **Tumor size** | <5cm | 21 | 11 | **0.012^*^** |
|  | >5cm | 11 | 21 |  |
| **Histologic** | Well | 1 | 2 | **0.013^*^** |
|  | Moderately | 14 | 7 |  |
|  | Poorly | 8 | 20 |  |
|  | Undifferentiated | 9 | 3 |  |
| **Lymphatic metastasis** | NO | 22 | 12 | **0.023^*^** |
|  | YES | 10 | 20 |  |
| **Distant metastasis** | NO | 32 | 28 | **0.113** |
|  | YES | 0 | 4 |  |
| **TNM stages** | I | 4 | 1 | **0.036^*^** |
|  | II | 14 | 7 |  |
|  | III | 14 | 20 |  |
|  | IV | 0 | 4 |  |

* Overall P<0.05
