# Supplementary material for: Over-expression of oncigenic pesudogene DUXAP10 promotes cell proliferation and invasion by regulating LATS1 and β-catenin in gastric cancer
Source: J Exp Clin Cancer Res. 2018 Jan 27;37:13. doi: 10.1186/s13046-018-0684-8 (PMC5787324; doi:10.1186/s13046-018-0684-8)
Supplement: Supplementary file 4 — Univariate and multivariate analysis of over-survival in gastric cancer patients (n = 64). (DOC 38 kb) [file 13046_2018_684_MOESM4_ESM.doc]

**Table S4** Univariate and multivariate analysis of over-survival in gastric cancer patients (n=64)

| **Variables** | **Univariate analysis** | | | **Multivariate analysis** | | |
| --- | --- | --- | --- | --- | --- | --- |
|  | **HR** | **95% CI** | **p value** | **HR** | **95% CI** | **p value** |
| age | 0.712 | 0.349-1.454 | 0.351 |  |  |  |
| gender | 1.036 | 0.516-2.081 | 0.921 |  |  |  |
| Histological subtype | 0.892 | 0.599-1.327 | 0.573 |  |  |  |
| Distribution | 1.073 | 0.666-1.729 | 0.773 |  |  |  |
| Lymph node metastasis(No vs .Yes) | 1.442 | 0.718-2.897 | 0.304 |  |  |  |
| Depth of cancer invasion | 0.867 | 0.734-1.444 | 0.867 |  |  |  |
| Tumor size | 2.096 | 1.031-4.263 | 0.041 | 1.509 | 0.708-3.215 | 0.286 |
| Distant metastasis | 4.733 | 1.245-18.000 | 0.023* | 4.450 | 1.080-18.339 | 0.039* |
| TNM stage (IIIa vs. I or II) | 2.834 | 1.472-5.454 | 0.002* | 2.276 | 1.187-4.364 | 0.013* |
| DUXAP10 expression(High vs.Low) | 3.357 | 1.609-7.003 | 0.001* | 2.404 | 1.068-5.411 | 0.034* |

HR, hazard ratio; 95 % CI, 95 % confidence interval, * Overall P < 0.05.
