# Supplementary figures and images for: Over-expression of oncigenic pesudogene DUXAP10 promotes cell proliferation and invasion by regulating LATS1 and β-catenin in gastric cancer
Source: J Exp Clin Cancer Res. 2018 Jan 27;37:13. doi: 10.1186/s13046-018-0684-8 (PMC5787324; doi:10.1186/s13046-018-0684-8)

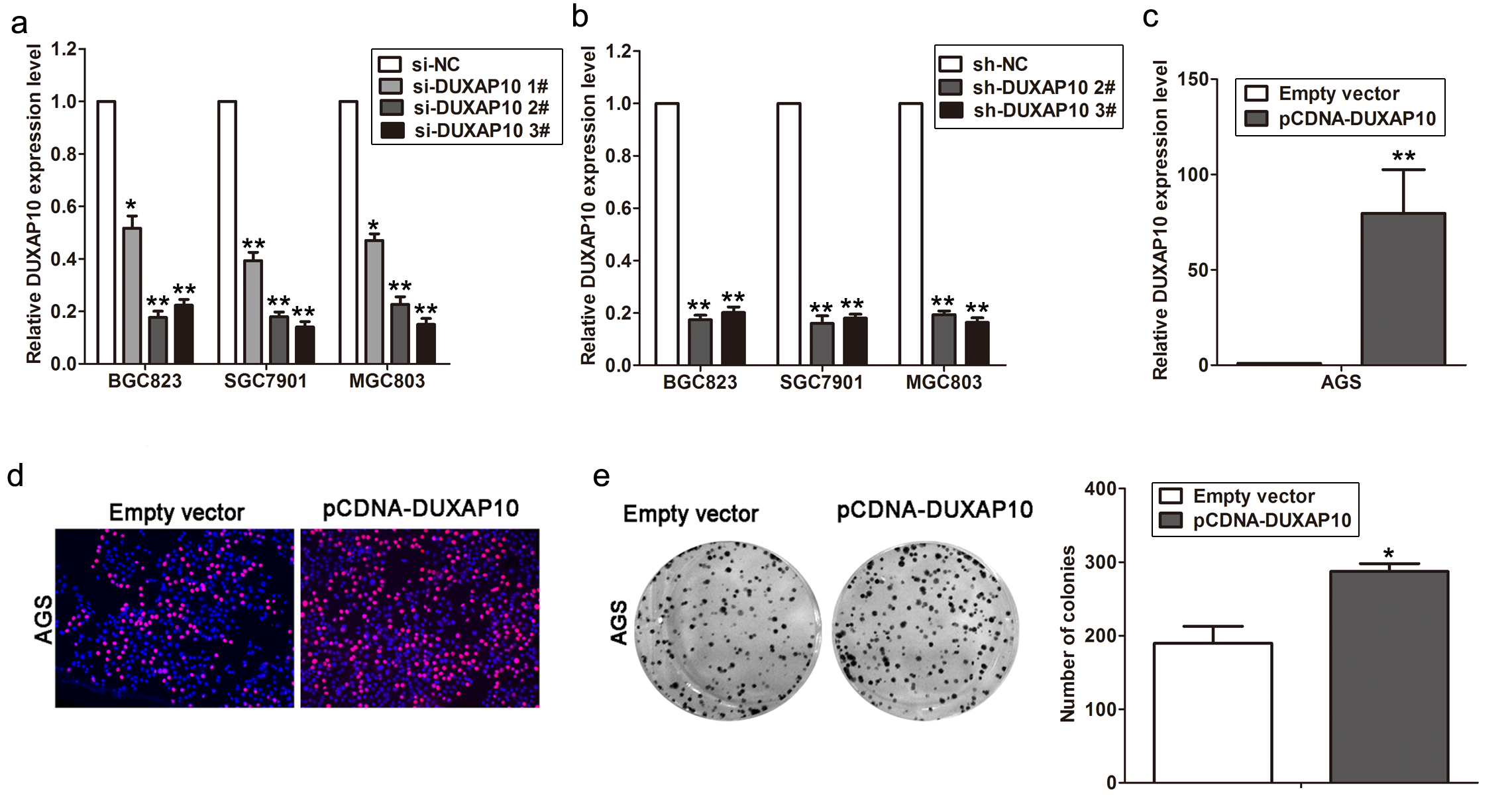

Supplement: Supplementary file 5 — (a, b) Analysis of the pseudogene DUXAP10 expression levels in BGC823, SGC7901, and MGC803 cells after transfection with DUXAP10 siRNAs or shRNAs by qPCR. (c) Analysis of DUXAP10 expression levels in AGS cells after transfection with DUXAP10 over-expression vector. (d, e) EdU incorporation and colony formation assays were performed to evaluate the effect of DUXAP10 over-expression on AGS cells proliferation. *P < 0.05 and **P < 0.01 (TIFF 9825 kb) [file 13046_2018_684_MOESM5_ESM.tif]

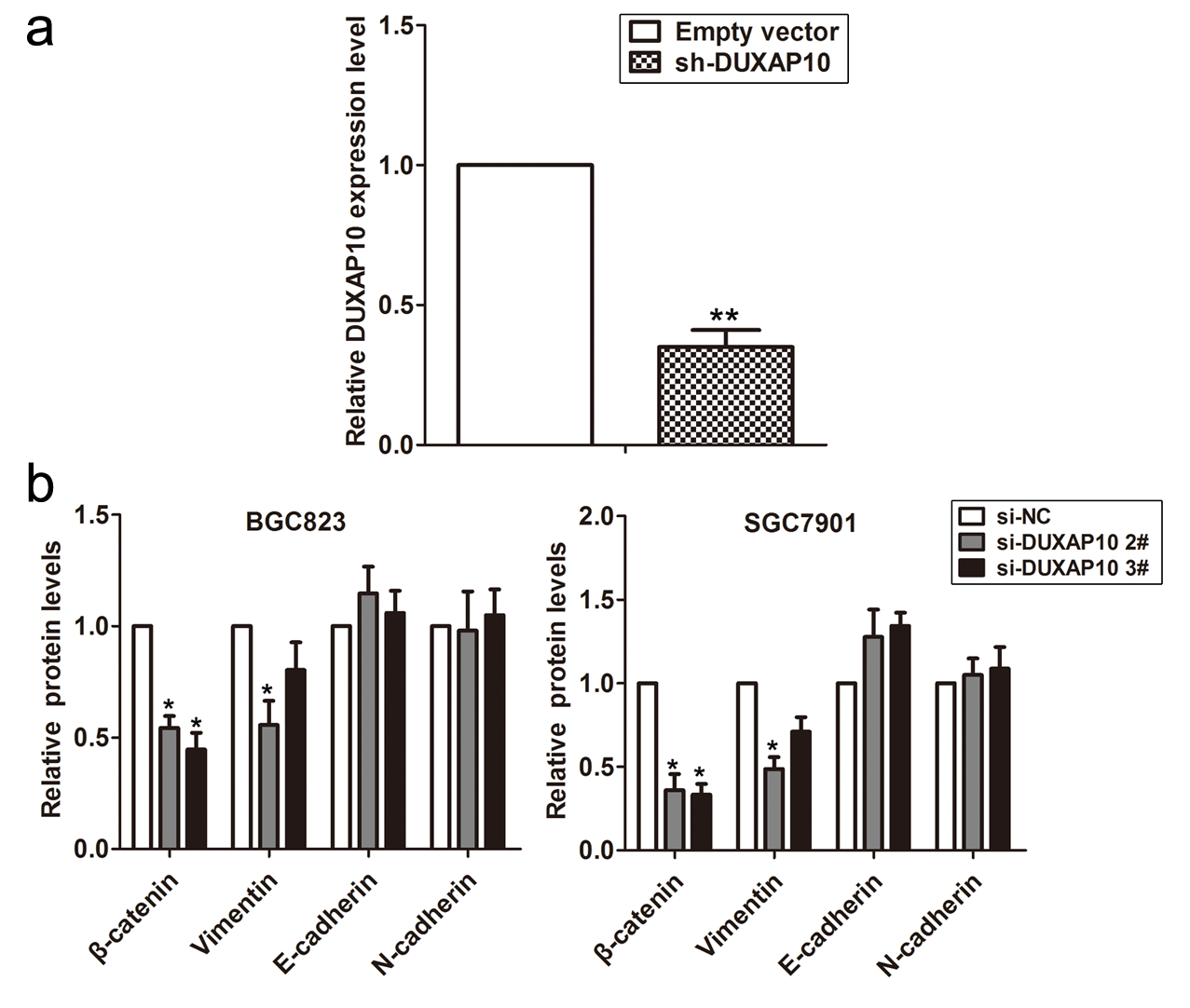

Supplement: Supplementary file 6 — (a) Analysis of DUXAP10 expression levels in tumor tissues collected from sh-DUXAP10 group and control group mice by qPCR. (b) Statistical analysis of E-cadherin, N-cadherin, Vimentin and β-catenin protein levels in DUXAP10 or negative control siRNAs transfected cells. *P < 0.05 and **P < 0.01 (TIFF 4831 kb) [file 13046_2018_684_MOESM6_ESM.tif]
